# A Case of Traumatic Catheterisation leading to Rectal Perforation and Periprostatic Abscess

**DOI:** 10.1155/2022/8656233

**Published:** 2022-06-15

**Authors:** Orla Cullivan, Silviu David, Syed Jaffry

**Affiliations:** Department of Urology, Galway University Hospital, Galway, Ireland

## Abstract

A 79-year-old gentleman presented to the Emergency Department (ED) with catheter-related issues on a background of a long-term catheter for previous urinary retention, Hartmann's procedure for colorectal cancer, and brachytherapy for prostate cancer. A 3-way silicone catheter was placed by ED staff and bladder irrigation commenced. The urine draining following catheterisation was found to be dark and thick, and irrigation fluid was noted to be draining per rectum. CT imaging was performed and demonstrated the catheter tip extending through the posterior wall of the urethra and into the rectum. The patient was admitted under the urology team, and urinary diversion was achieved with a suprapubic catheter. Subsequent imaging demonstrated a periprostatic abscess, which was initially managed with antimicrobial therapy, followed by attempted image-guided drainage. Repeat imaging following a 6-week course of antibiotics failed to show an improvement in the collection. During his inpatient stay, he contracted COVID-19 and passed away suddenly. This case demonstrates the potential catastrophic consequences associated with urethral catheterisation.

## 1. Introduction

Urinary catheterisation represents a relatively common healthcare procedure that can be associated with a variety of complications. This case explores a case of traumatic catheterisation and the resulting consequences for the patient and challenges for the treating healthcare team.

## 2. Case History

A 79-year-old gentleman presented to the Emergency Department (ED) reporting symptoms of bypassing from his urinary catheter, bleeding from his urethral meatus, and dark urine in his catheter bag. Four months prior to this attendance, he developed urinary retention requiring catheterisation. He subsequently failed two voiding trials. Past history included prostate cancer treated with brachytherapy 11 years ago, urethral stricture disease, and T4N1 colorectal cancer for which he underwent a Hartmann's procedure 4 years ago. Endoscopy of his rectal stump one month prior to his presentation demonstrated proctitis. The patient himself denied a history of passing urine per rectum. The patient had a normal flexible cystoscopy one year previously and a normal CTTAP 3 years previously.

**Figure 1:**
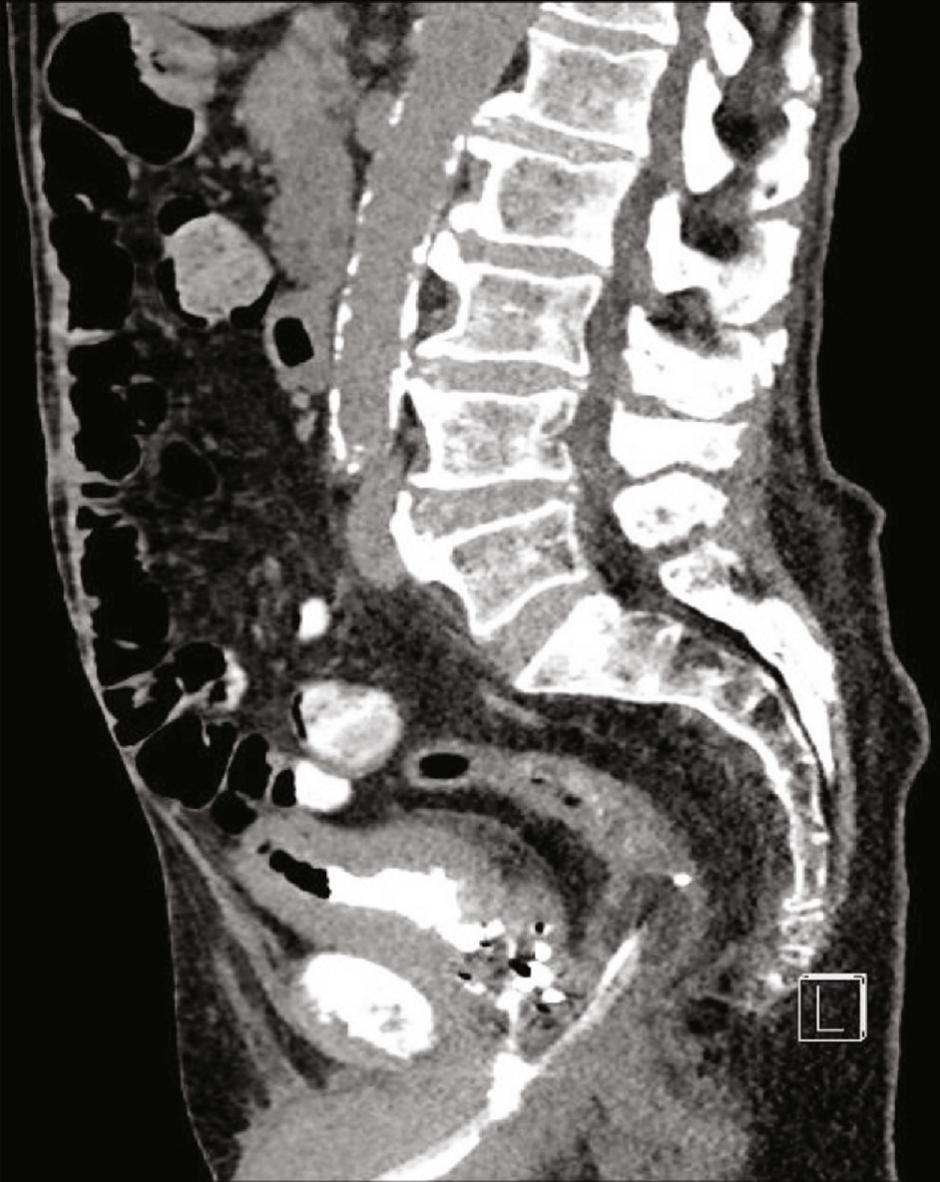
Sagittal CT demonstrating catheter placement in rectum.

**Figure 2:**
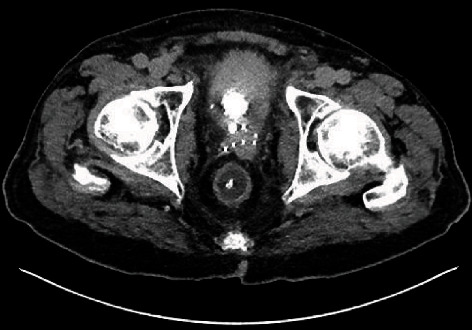
Axial CT demonstrating catheter placement in rectum.

The patient's indwelling catheter was changed to a 20 French 3-way catheter by ED staff, and bladder irrigation was commenced. Blood was noticed in the catheter bag immediately following catheterisation. Subsequently, the urine draining was described as thick and dark in appearance, and irrigation fluid was noted to be draining from the patient's rectum. Blood results demonstrated mildly raised inflammatory makers with white cells of 11.8 × 10^9^/L(RR 4-10 × 10^9^/L) and a C-Reactive Protein of 68 mg/L (RR 0-5 mg/L). Creatinine was at the patient's baseline of 130 *μ*mol/L (RR 64-104 *μ*mol/L). CT imaging demonstrated a malpositioned catheter with the catheter tip extending through the posterior wall of the prostatic urethra and into the rectum, creating a rectourethral fistula (Figures 1 and 2). The bladder was also noted to be thick-walled secondary to chronic outflow obstruction and radiotherapy.

**Figure 3:**
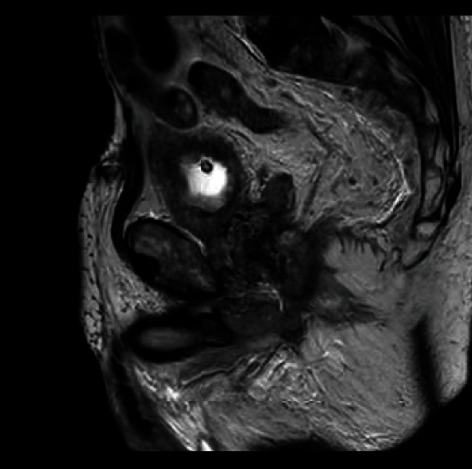
Sagittal MRI of periprostatic collection.

**Figure 4:**
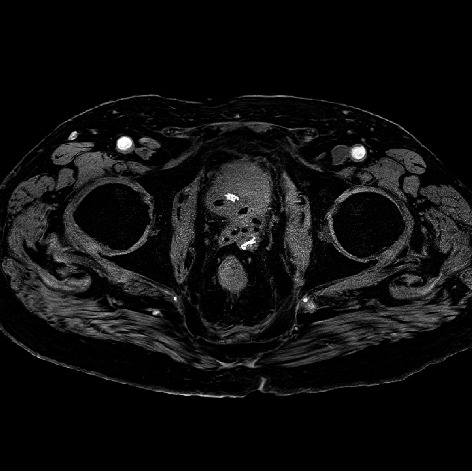
Axial CT of periprostatic collection.

The patient was admitted under the urology team, and urinary diversion was achieved with a suprapubic catheter. He was reviewed by the colorectal team, who were happy that no surgical intervention was needed as an inpatient, and would arrange outpatient follow-up. The patient reported persistent rectal pain, so pelvic MRI imaging was arranged. This demonstrated an extensive pelvic abscess involving the prostate with concerns for early osteomyelitis of the inferior left pubic ramus (Figures 3 and 4). Urine cultures grew Klebsiella pneumonia. The patient was initially treated with Augmentin, then switched to Piperacillin-Tazobactam on the advice of microbiology colleagues due to rising inflammatory markers. Inflammatory markers failed to improve following 10 days of this, and treatment was changed to oral Ciprofloxacin and Metronidazole, with a 6-week total course advised.

**Figure 5:**
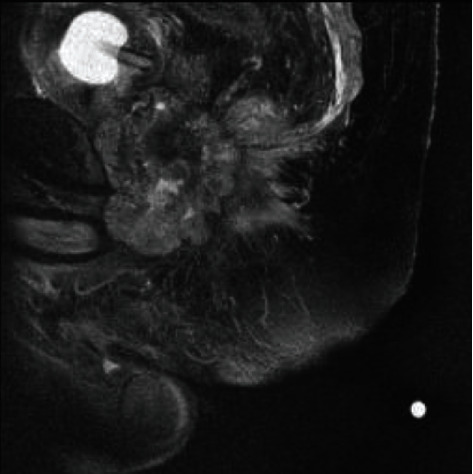
Saggital MRI imaging demonstrating persistence of collection post-antibiotic treatment.

**Figure 6:**
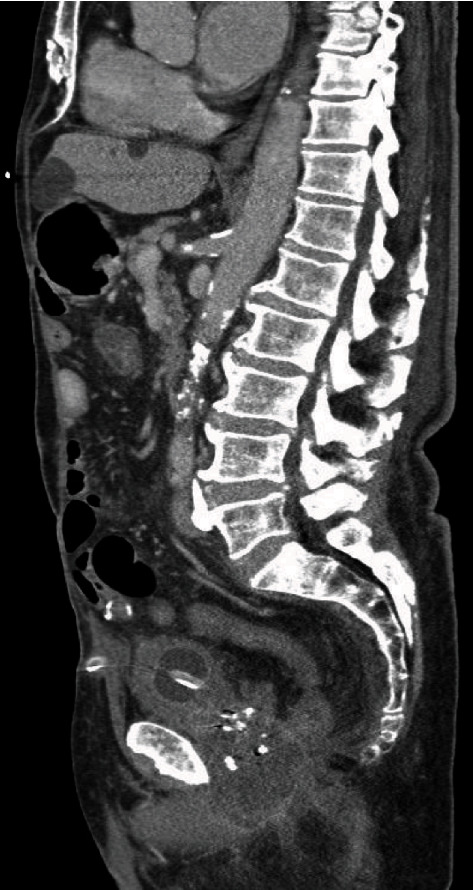
Saggital CT imaging demonstrating persistence of collection post antibiotic treatment.

The patient remained in the hospital to complete his antibiotic course and became acutely unwell approximately 5 weeks into his admission. He was reviewed by the medical, intensive care, and microbiology teams. The impression was that the patient had a septic event related to his abscess. CT pulmonary angiogram and COVID-19 tests were performed and were both negative. There was no growth on repeat urine and blood cultures. Antibiotics were changed to Meropenem, Vancomycin, and Daptomycin. CT TAP was performed which demonstrated thickening of the wall of the transverse colon. A repeat MRI pelvis was obtained which demonstrated an unchanged pelvic abscess (Figures 5 and 6). The patient was then arranged for transrectal image-guided drainage of the abscess with interventional radiology. A small amount of blood-stained material was aspirated, and a 10Fr drain was sited, but it was deemed that the collection was mostly solid and necrotic therefore not amenable to drainage. Enterococcus faecium sensitive to Vancomycin was isolated from the aspirate. The drain dislodged 2 days postinsertion and was not replaced as the preceding output had been minimal.

The patient contracted COVID-19 during his stay and was taken over by the Infectious Diseases team as per the hospital policy. He developed a hypoactive delirium secondary to his COVID-19 infection and subsequently passed away, likely due to COVID-19-related complications.

## 3. Discussion

Urinary catheterisation is a common intervention performed for a variety of reasons including urinary retention, monitoring of urinary output in critically ill patients and selected surgical cases, and in prolonged immobilisation [1]. Although there is variation in the figures reported in the literature, it is estimated that approximately 25% of patients admitted to hospital have a urinary catheter during their inpatient stay [2]. A UK study performed in 2017 found that 18.6% of hospital inpatients had a urinary catheter at any one time [3]. Despite the relative ubiquity of catheterisation as an intervention, there are several complications associated with this process, including infection, trauma, malignancy, hypersensitivity, blockage, and fistula formation [4, 5].

It has been recorded that genitourinary trauma due to catheterisation occurs in 1.5% of catheter days [5]. A review of iatrogenic urethral catheterisation injuries performed across two Irish tertiary centres calculated the total cost of managing 37 injuries over a 6 month period as €335,377 [6]. Clearly, urethral catheterisation represents a significant burden of cost and morbidity. Dellimore et al. [4] describe how severe urethral trauma, which includes perforation, is directly related to mechanical interaction between the catheter and urethra, with additional catheter characteristics such as shape and physical properties as possible contributing factors. As a potential strategy to combat this, they recommend alterations to catheter manufacturing to produce catheters that have a hydrophyllic, antibacterial, low-friction, and durable coating, with a less-rigid tip, stiff shaft, and a structure that allows minimal contact between the catheter and urethra [4].

Rectal perforation is indeed a rare complication of urethral catheter insertion, with few previous cases reported in the literature [7]. Therefore, little is known about such an occurrence. We attribute the cause of our patient's rectal perforation to his previous brachytherapy treatment and known proctitis. Brachytherapy is a common treatment choice for localised prostate cancer [8]. However, it is associated with several adverse effects including radiation proctitis, sexual dysfunction, incontinence, urethral stricture, and haematuria [9]. Rectourethral fistula is also a recognised complication of brachytherapy treatment [10]. However, given that this patient had a recent endoscopy which only demonstrated proctitis and denied a history of passing urine per rectum, it is unlikely that he had a preceding rectourethral fistula. Radiation proctitis can induce tissue changes of endarteritis, inflammation, and fibrosis [11, 12]. This can result in friable and ulcerated tissue, which can be more susceptible to injury, as happened to this patient. This case is a cautionary tale about the potential harms that can result from what is considered a common and relatively straightforward procedure.
